# Supplementary material for: Mirror effect in atomic force microscopy profiles enables tip reconstruction
Source: Sci Rep. 2020 Nov 3;10:18911. doi: 10.1038/s41598-020-75785-0 (PMC7641199; doi:10.1038/s41598-020-75785-0)
Supplement: Supplementary file 1 — Supplementary Information. [file 41598_2020_75785_MOESM1_ESM.pdf]

Supplementary Information:

Mirror Effect in Atomic Force Microscopy Profiles Enables Tip Reconstruction

Francisco Marqués-Moros, Alicia Forment-Aliaga, Elena Pinilla-Cienfuegos, and Josep Canet-Ferrer*

[1] Instituto de ciencia molecular (ICMol), Universidad de Valencia, Paterna, Spain
E-mail: jose.canet-ferrer@uv.es

SI1. Graphical tip reconstruction with a narrow rectangular object.

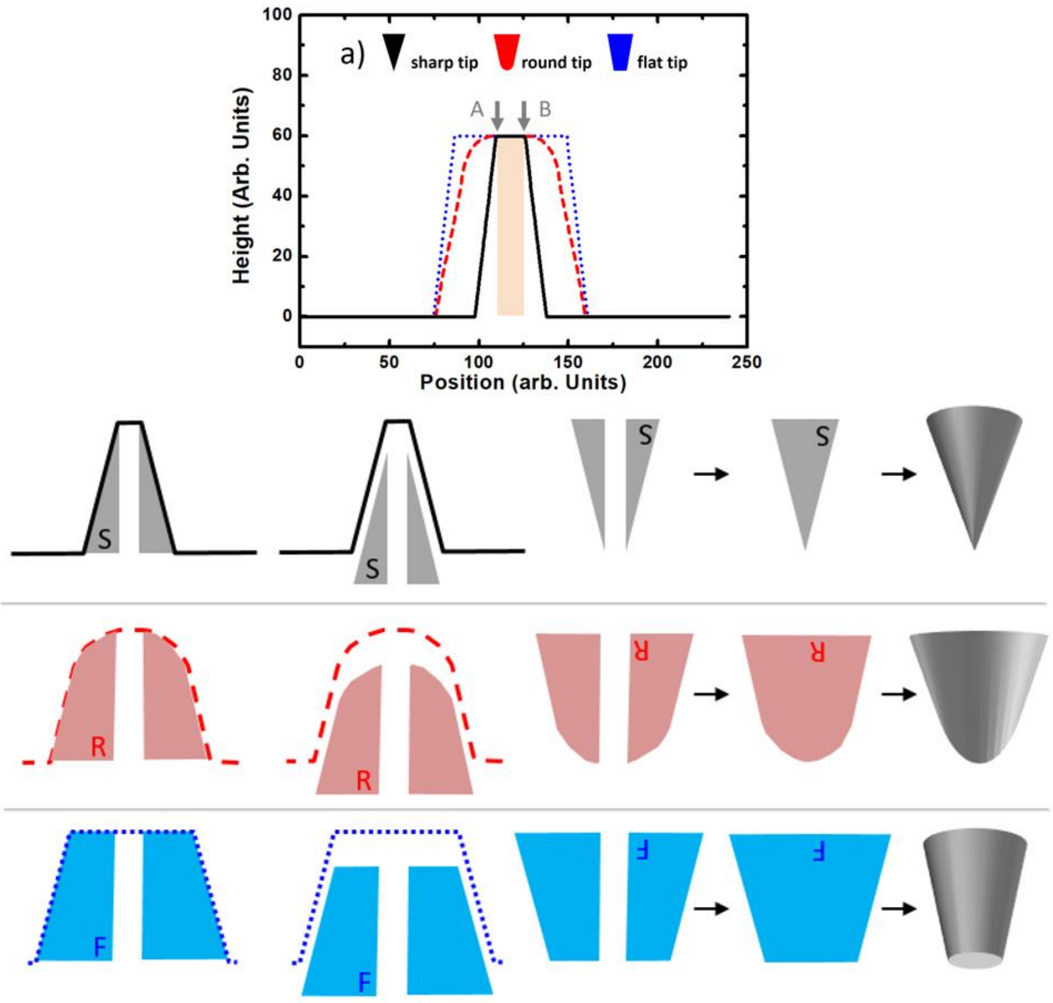

Figure SI1. Schematic representation of the tip reconstruction based on the mirror effect. It is presented step by step, showing how the corresponding tip-shape is obtained from he numerically simulated profiles (S for sharp tip; R for round tip; F for flat tip).

SI2. Profiles processed in the manuscript and steps done for tip reconstruction.

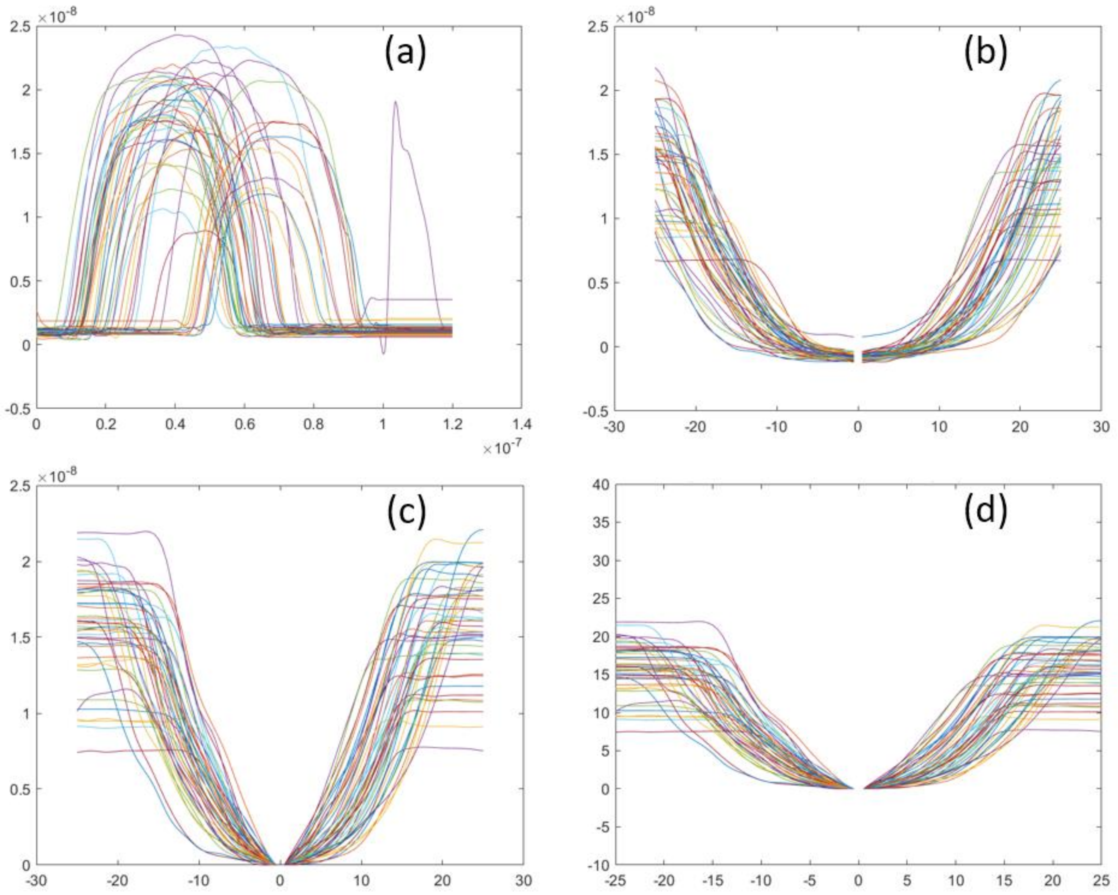

Figure SI2. Tip profile plots processed by means of a sequential algorithm in order to analyze numerically and average all the measured profiles. (a) The 46 profiles are interpolated to the same values in the abscissa (position) for an easier processing; then, they are inverted and aligned to the respective maxima (b). (c) The profiles after width correction –half-a-width of the height is removed in both sides–. (d) Rescaled profiles with units converted from meters to nanometers. Eventually, in these plots we can find some artifacts related with the acquisition of non-symmetric profiles (profiles with an odd number of data points) or sample tilt shifting the maxima from the center.

SI3. Algorithm to process the experimental profiles for tip reconstruction.

In order to ensure an objective assessment of the tip reconstruction, the data are processed by means of the algorithm below.

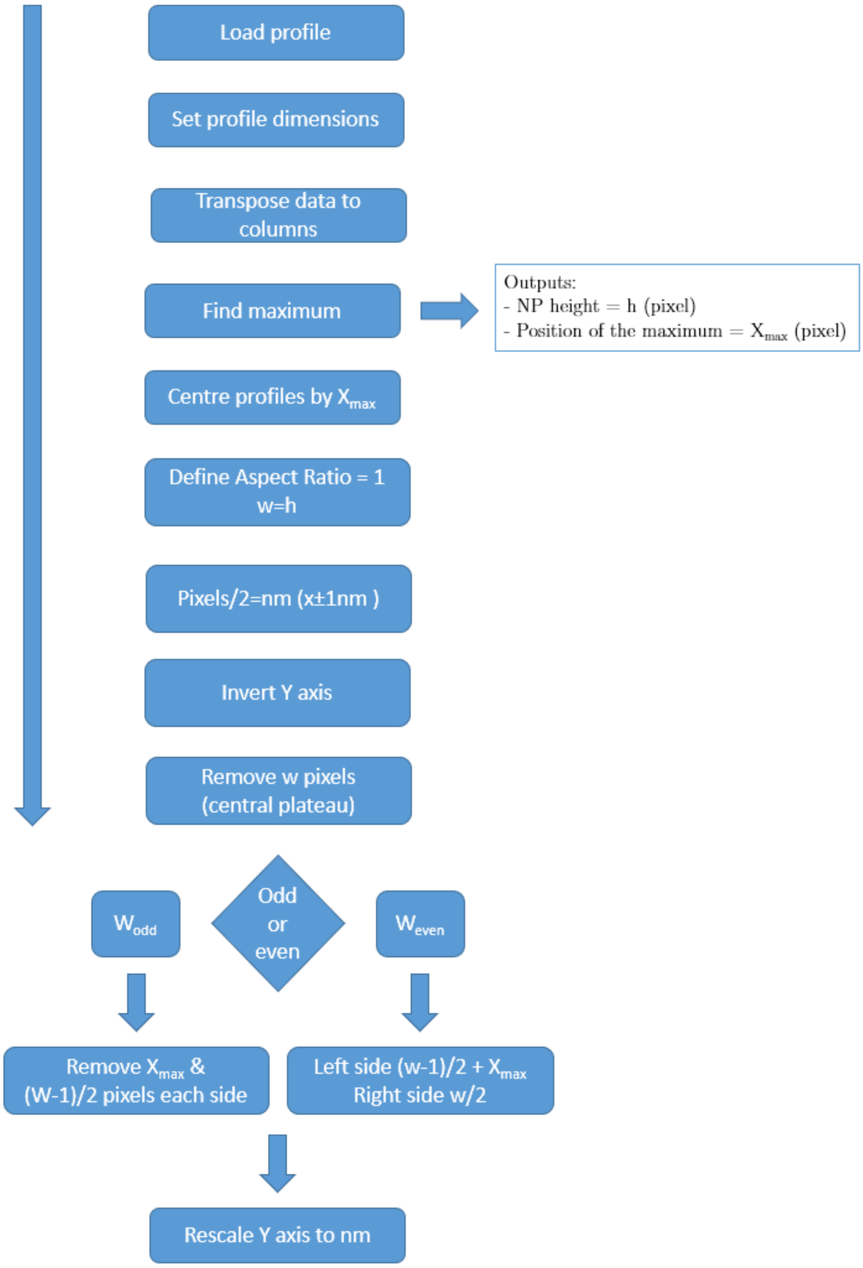

Figure SI3. The script uploads all the profiles as variables and it interpolates them for easing parallel processing, the unit of the profiles are pixels most of the time. Afterwards, the maximum of every profile is identified, so they can be ordered with respect to the maxima (considered as center of the profile). Then, the real width of the object is determined according to the aspect ratio to estimate the object width (w = h for cubic objects). After that, the script rounds the height (in pixels) to remove half-a-width pixels in both sides of the profiles. To conclude, the profiles are re-scaled and *y* variables are converted to nanometers.

SI4. Artefacts at the experimental profiles.

When applying the h-w curve approach to a real case, the experimental tip shape profiles might present irregularities. For example, in the case cubic NPs we have observed several profiles exhibiting asymmetric shape, despite of the symmetry of NPs. These asymmetries can appear for several reasons such as sample tilt, scanning artefacts or defects on the NPs.

In Fig. SI4(a), we show an example of the proper tip reconstruction from a considerably symmetric profile. In Fig. SI4(b), we show a profile with a noticeable protrusion in the tip, which avoids an accurate reconstruction. In Fig. SI2(c), the maximum of the profile is not at the centre of the tip. In those cases, it is not possible to identify the proper tip shape. The accuracy of our results could be improved just by removing irregular profiles [like (b) and (c)] from the statistics, but we do include them to show the robustness of the method. From our results we can conclude that a certain error in height measurement is assumable. In the next section we will give some details.

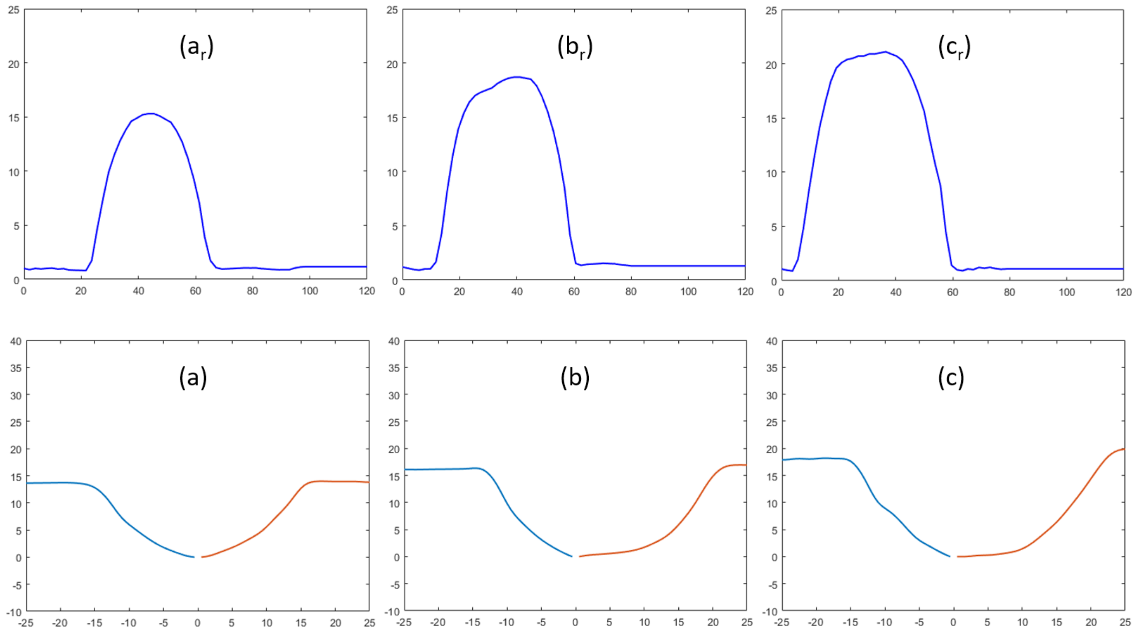

Figure SI4. Tip reconstruction for three different profiles used in the manuscript. (a_r) corresponds to the row data of a symmetric profile, but eventually we can find asymmetric profiles like (b_r, c_r). (a) Those artifacts have a minor impact in the h-w curve, however they might affect the tip reconstruction, as shown in (b) and (c).

SI5. Height-to-width (and width-to-height) fitting.

SI5.1 Object larger than the tip radius.

As described in the manuscript, the convolution error can be estimated from the w-h relation for rectangular motifs larger than the tip radius, by using the expressions:

$$\frac{1}{2}w_{exp} = r_{tip} + \Delta + \frac{1}{2}w \qquad (1)$$

$$\Delta = (h - r_{tip})\, tan(\gamma) \qquad (2)$$

being w_{exp} the experimental width profile, w the real width and h the height of the object under inspection. The convolution effects are the sum of the tip radius (r_{tip}) and Δ which for objects larger than the tip radius accounts for the contribution of the tip-to-face angle (γ). Working with cubic objects we can consider $w=h$, and hence, Eq. (2) can be substituted in Eq. (1) to fit the resulting expression to the experimental curve. As a result r_{tip} and $tan(\gamma)$ are obtained as fitting parameters, as done in Fig. 4(c):

$$\frac{1}{2}w_{exp} = r_{tip} + (h - r_{tip})\, tan(\gamma) + \frac{1}{2}h \qquad (3)$$

Eventually, the reader could prefer to develop Eq. (3) to obtain r_{tip} and $tan(\gamma)$ from a linear regression as follow:

$$w_{exp} = 2r_{tip} + 2(h - r_{tip})\, tan(\gamma) + \mathrm{h} \qquad (4)$$

$$w_{exp} = 2r_{tip} + 2\, htan(\gamma) - 2r_{tip}\, tan(\gamma) + \mathrm{h} \qquad (5)$$

$$w_{exp} = 2r_{tip} * [1 - tan(\gamma)] + h * [2\, tan(\gamma) + 1] \qquad (6)$$

So, the dependence of h on w_{exp}

$$h = \frac{w_{exp}}{[2tan(\gamma)+1]} - \frac{2r_{tip*}(1-\tan(\gamma))}{[2tan(\gamma)+1]} \qquad (7)$$

Where the slope is = $\frac{1}{[2tan(\gamma)+1]}$ (9)

Using the data from Fig. 4 we would find h = *0,5924*w_{exp} - *11,703* nm which leads to γ = 19.0 degrees and r_{tip} = 15.1 nm. As discussed in the manuscript the determination of r_{tip} by means of h-w method is affected by the error in the determination of $tan(\gamma)$, see Eq. (9).

SI5.2 Tip radius larger than the object height.

In general, the object size will be shorter than the tip radius when using metal coated probes, such as conductive or MFM tips. Of course, we can use analogous method, however, the expressions would be rather different because in this operation range:

$$\frac{1}{2} w_{exp} = \Delta + \frac{1}{2} \mathrm{w} \quad (9)$$

$$\Delta = r_{tip} * Cos\left[ArcSin\left(\frac{r_{tip}-h}{r_{tip}}\right)\right] \quad (10)$$

Developing Eq. (10) by squares we have that:

$$\Delta^2 = r_{tip}^2 * \left[1 - \left(\frac{r_{tip}-\mathrm{h}}{r_{tip}}\right)^2\right] \quad (11)$$

$$\Delta^2 = r_{tip}^2 - r_{tip}^2 \left(\frac{r_{tip}^2 + h^2 - 2r_{tip} * h}{r_{tip}^2}\right) \quad (12)$$

$$\Delta^2 = r_{tip}^2 - r_{tip}^2 - h^2 + 2r_{tip} * h \quad (13)$$

$$\Delta^2 = 2r_{tip} * h - h^2 \quad (14)$$

Considering w=h (as applies for cubic objects) and introducing Eq. (14) in Eq. (9),

$$w_{exp} = h + 2\sqrt{2h\, r_{tip} - h^2} \quad (15)$$

In this case, we can plot w-h to determine the tip as a fitting parameter to Eq. (15). Obviously, we cannot get information about the tip-to-face angle in this operation range. In Fig. SI5 we show the w-h curve for a MFM tip. The corresponding tip reconstruction is carried out in Fig. SI6.

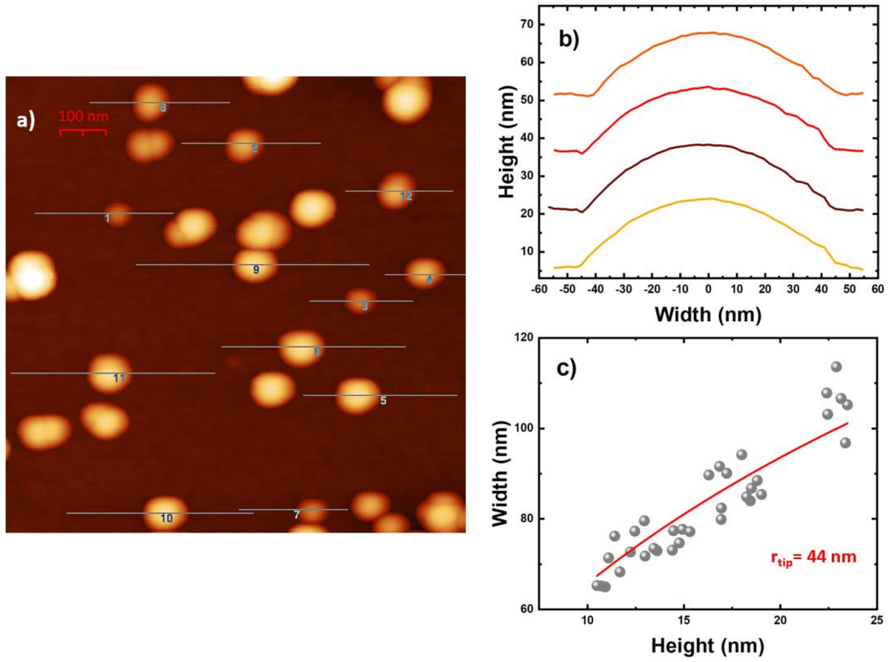

Figure SI5. W-h curve for a MFM tip, analogous to Fig. 4 found in the manuscript.

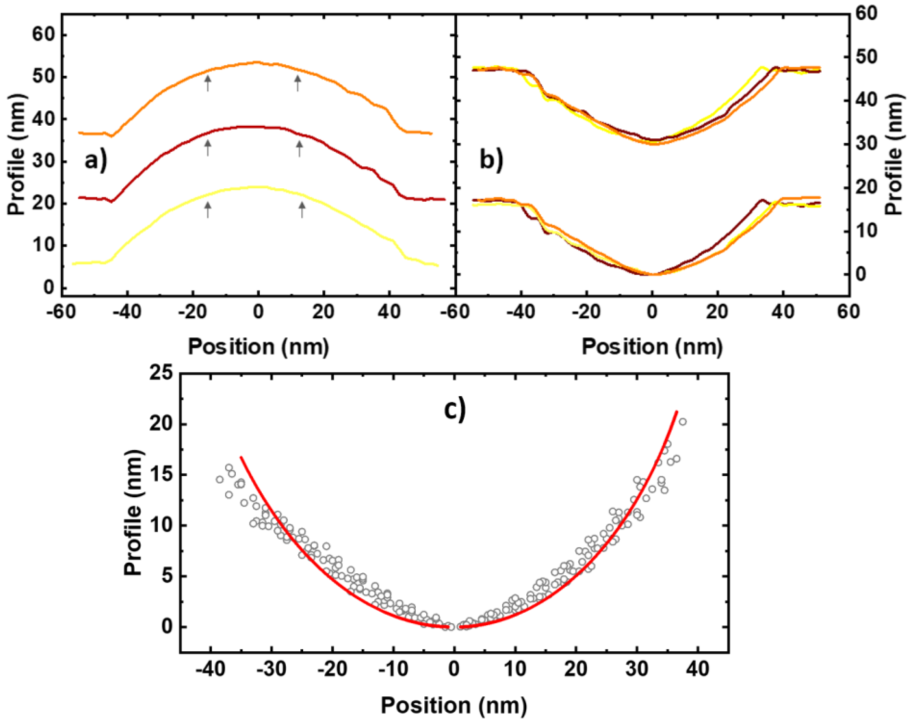

Figure SI6. Tip reconstruction of a MFM tip, analogous to Fig. 5 found in the manuscript.

SI6. Impact of the experimental error on the estimation of the tip parameters.

The NPs are randomly oriented on the substrate surface so we cannot be sure that the scan is aligned in parallel to the NP faces, indeed, it is likely to scan some of the profiles with the tip sliding diagonally to the NP.

This drives to a systematic error consisting of an overestimation in the measurement of the experimental profile, w_{exp}. In the worst case scenario, this error will have a maximum value of √2*h. In this section, we will show that this deviation might affect the values of r_{tip} during the fitting of the w-h curve, however, the impact in the case of the mirror effect is not so important.

It is worth noting that the AFM images present accuracy only in the height measurement. In contrast, the error in the lateral resolution can be in the order of 300 % for tinny objects. In the particular case of cubic NPs of 20 nm height the situation is the next:

$$\varepsilon\left(w_{exp}\right) = \sqrt{\varepsilon(\text{discretization})^2 + \varepsilon(\text{aspect ratio})^2 + \varepsilon(\text{random angle})^2 + \varepsilon(\text{scan})^2}$$

ε(discretization) corresponds to the pixels size, ε(aspect ratio) is estimated around 10 % from structural characterization by TEM, ε(random angle) will have a maximum value of about 40% so it must be in the order of 20% in average. Finally, ε(scan) could reach 50% in some cases.

On the other hand, we have learnt that these NPs tend to aggregate with certain order such a way that the aggregates look like large NPs in the AFM images. For this reason, we focus on isolated NPs with regular shape profiles. With this strategy, we have unintentionally reduced the contribution of those profiles acquired along the diagonal on the cubic NPs as these would show an elongated shape. As the same time we are also preventing the contribution of those NPs affected by scanning artefacts.

Despite this effort, the results from the two approaches proposed on the manuscript differ. While the r_{tip} is overestimated using the w-h plot approach, it is accurately estimated by the mirror effect approach, and it seems the other way around for the tip-to-face angle. The reasons have to do with the way the data are managed:

In the case of the w-h curve, tan(γ) is obtained from the slope with the accuracy of averaging 46 profiles, see Eq. 7 from supporting information. However, r_{tip} depends on both the slope and the intercept, and unfortunately, it is deduced from a more complicated expression. Therefore, it is exposed to a huge error propagation. See the error bars in the figure below, the dispersion in the abscissa will affect the slope but taking a large number of points this dispersion can be compensated. However, the intercept will be overestimated in any case.

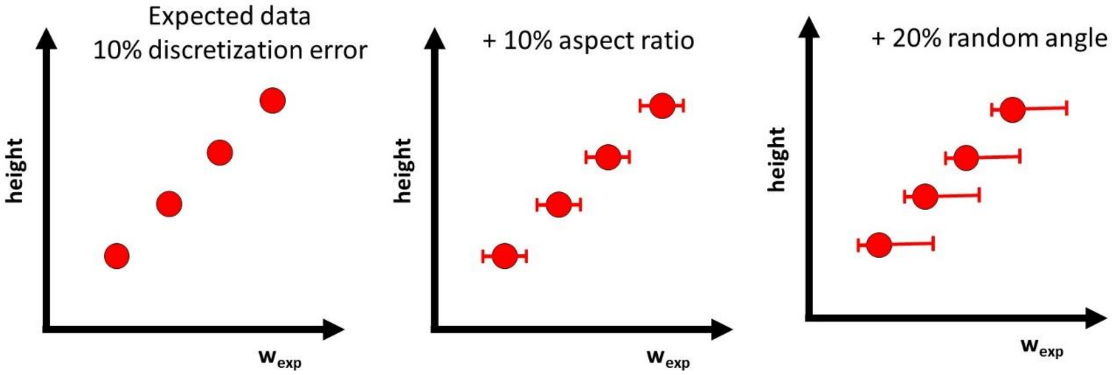

In the case the mirror effect, we can obtain a plot of the tip from every profile. As a result, deviations on the estimation of r_{tip} compensate each other giving a more accurate average value. Indeed, the shape and size of the tip is clearly reproduced in the scatter plot. With the fitting we

finally quantify, giving values to the tip depicted in the plot, i.e. Fig. 5c. As a result, we can conclude than in proposed experimental conditions the error in w_{exp} has a minor impact in the fitting, because in general AFM does not required high lateral resolution. In this sense, those NPs driving to an overestimation in w_{exp}, for example due to a deviation about 50% in the aspect ratio, could be easily identified as their profile will not fit with a round tip.

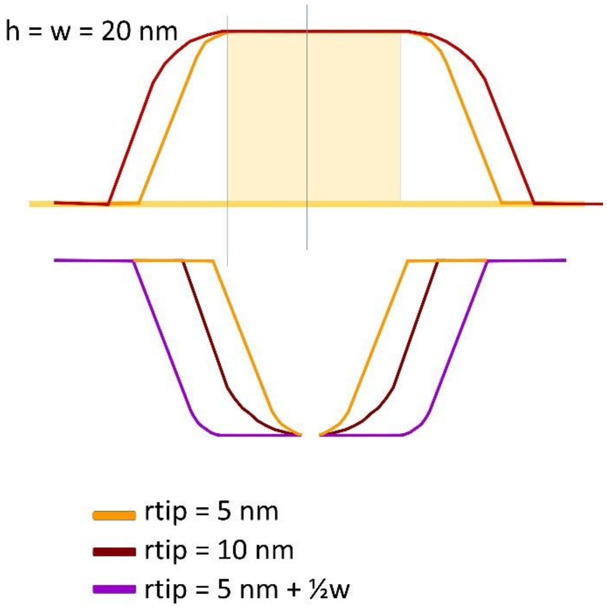

Finally, have done some statistics to discuss semi-quantitatively our qualitative discussion above and it turns out that the corresponding deviation is assumable if compared with the total experimental error, and importantly, that both methods are complementary. The statistics is done using 32 profiles out of the 46 used in the manuscript images (notice that Eq. 2 does not apply in case of $h < r_{tip}$ so we are limited to profiles with h > 15 nm). From the experimental profiles we can obtain h and w_{exp}. Then, we use Eqs. 1 and 2 to calculate w and Δ respectively. According our results in Figs. 4 and 5 of the manuscript, we considered the next sets of parameters [r_{tip} = 15 nm and γ = 19º] and [r_{tip} = 7 nm and γ = 20º].

	h (nm)	w_{exp} (nm)	Δ (nm) r_{tip} = 15 nm	w (nm) r_{tip} = 15 nm	Δ (nm) r_{tip} = 7 nm	w (nm) r_{tip} = 7 nm
**Average**	**18.8**	**51.2**	**1.1**	**18.3**	**4.0**	**29.0**

The average height of the 32 NPs under study is 18.8 nm giving an average w_{exp} = 51.2. Considering r_{tip} = 15 as estimated from the h-w curve we obtain w = 18.3, this is h ~ w. While w ~ 1.54 h (relative error of about 54%) if we consider r_{tip} = 7 nm as obtained by means of the mirror effect. As we wanted to demonstrate, the w-h curve deviates the values of r_{tip} in order to satisfy Eqs. 1 and 2. In contrast, in the case of the mirror effect we can obtain a good estimation of the tip parameters despite the error in the lateral resolution, in this case accumulating the error in the tip-to-face angle.
